# The Navigate First Psychosis Program: A balance between the medical and recovery models in the debate about long term prophylactic antipsychotics. Mission Impossible?

**DOI:** 10.1192/j.eurpsy.2022.2056

**Published:** 2022-09-01

**Authors:** L. Sharony, R. Josman

**Affiliations:** Mazor Mental Health Center, Psychiatry Public Clinique, Acre, Israel

**Keywords:** Navigate, long term antipsychotic treatment, shared decision making, first psychosis

## Abstract

**Introduction:**

First psychosis programs have been developed during the past 30 years to influence the prognosis of a first psychotic episode by early integrative biopsychosocial interventions, with a focus on the processes that contribute to relapse. In the process of recovery, Navigate program emphasis on enabling a connection to what is important to the person (work, studies, relationships, intimacy), thus strengthening resiliency and quality of life and reducing self-stigma. Medication is part of any intervention program, however, there is a lot of ambivalence amongst the young person and family about its continuation and many will stop the medication altogether. Moreover, although evidence for the benefits of antipsychotic medication in short-term treatment is well established, there is an ongoing debate in the professional medical literature about the need and benefit of routine prophylactic long-term antipsychotics after first psychotic episode. There is also a significant uncertainty concerning the proportion of patients that will maintain remission without antipsychotics.

**Objectives:**

In this lecture, we will present some of the lessons that we have learned and are still learning from our clients, together with case examples.

**Methods:**

In our Navigate Program, we have developed strategies based on literature and experience that enables the person/family to be part of the decision-making process, which at times presents dilemmas and risks but also promotes the potential for growth and transformation.

**Results:**

How do we talk about the medication issue? Who can continue without medication or with very low dosage? How can we taper antipsychotic treatment?

**Conclusions:**

Are we willing to take the risk?

**Disclosure:**

No significant relationships.

